# Women's perspective on life after total laryngectomy: a qualitative study

**DOI:** 10.1111/1460-6984.12511

**Published:** 2019-11-01

**Authors:** Klaske E. van Sluis, Anne F. Kornman, Lisette van der Molen, Michiel W. M. van den Brekel, Gili Yaron

**Affiliations:** ^1^ Department of Head and Neck Oncology and Surgery Netherlands Cancer Institute‐Antoni van Leeuwenhoek Amsterdam the Netherlands; ^2^ Amsterdam Center for Language and Communication University of Amsterdam Amsterdam the Netherlands; ^3^ Department of Health Services Research, Care and Public Health Research Institute, Faculty of Health, Medicine and Life Sciences Maastricht University Maastricht the Netherlands

**Keywords:** total laryngectomy, functional outcome, stigma, quality of life, women, qualitative

## Abstract

**Background:**

Physical and psychosocial challenges are common after total laryngectomy. The surgery leads to lifelong changes in communication, airway, swallowing and appearance. As we move towards health models driven by patient‐centred care, understanding the differential impacts of surgical procedures on subgroups of patients can help improve our care models, patient education and support systems. This paper discusses the experiences of women following total laryngectomy.

**Aims:**

To gain an insight into the impact of total laryngectomy on women's daily life while identifying their specific rehabilitation needs.

**Methods & Procedures:**

This paper is based on in‐depth, semi‐structured interviews with eight women who had undergone total laryngectomy. These interviews were conducted with women at least 1 year after they had undergone total laryngectomy, and the participants did not have recurrent disease. Using an interview guide, participants were encouraged to discuss their everyday experiences, while also focusing on issues typical to women. The transcribed interview data were analysed by thematic analysis, taking interpretative phenomenological analysis as a lead.

**Outcomes & Results:**

The interviews revealed three main themes: disease and treatment as a turning point, re‐establishing meaningful everyday activities, and persistent vulnerability. Participants reported experiencing challenges in their rehabilitation process due to physical disabilities, dependency on others and experienced stigma. Women‐specific challenges arose in dealing with the altered appearance and voice, performing care activities, and the spousal relationship (including intimacy).

**Conclusions & Implications:**

Women who undergo total laryngectomy are likely to experience issues in returning to work, the performance of informal care‐work, the spousal relationship, intimacy and social interaction due to stigmatization. Medical pretreatment counselling and multidisciplinary rehabilitation programmes should help patients form realistic expectations and prepare them for the changes they will face. A gender‐ and age‐matched laryngectomized patient visitor can contribute to this process. Rehabilitation programmes should incorporate the partner and offer psychosocial support for women following total laryngectomy to return to their former roles in family life, social life and work‐related activities.

**What this paper adds**

**What is already known on the subject**

Total laryngectomy is a major surgery that has a significant impact on the affected individuals’ quality of life. Patients are confronted with long‐term consequences including communication problems, altered breathing, swallowing issues, changed appearance and psychosocial issues. The current, limited body of qualitative literature on the impact of total laryngectomy predominantly features the perspective of men. As a result, little is known about experiences specific to women.

**What this paper adds to existing knowledge**

Using a qualitative approach, this paper shows that women after total laryngectomy experience problems in returning to work, the performance of informal care‐work, the spousal relationship and spousal intimacy. Women dislike their new voice and experience stigma due to their unusual voice and appearance. This study demonstrates the value of peer support in pretreatment counselling and rehabilitation, especially with gender‐ and age‐matched individuals.

**What are the potential or actual clinical implications of this work?**

To prepare female patients undergoing total laryngectomy adequately, they should receive counselling on the impact of the procedure on their everyday life. A gender‐ and age‐matched laryngectomized patient visitor can contribute to this process. The provided counselling and support should involve the patient's partner so that the impact of the surgery on the relationship and family roles can be discussed. This will allow these women to form realistic expectations, prepare them for the changes they will face and help them reintegrate into their former roles in family life, social life and working towards work‐related activities.

## Introduction

Total laryngectomy has major consequences for essential physical functions including airway, swallowing and speech. With the removal of the larynx, the natural voice is lost, and patients have to rehabilitate speech, often with help of a voice prosthesis (van der Molen *et al*. 2013). In addition, patients also have a changed appearance due to the creation of a permanent tracheostoma. The physical alterations lead to ongoing functional issues including reduced physical fitness, problems with food intake, frequent coughing and communication problems. As a result, patients may be confronted with psychosocial issues (Bickford *et al*. 2018, Perry *et al*. 2015).

Since survival and complication rates improved over the last decades, scholarly attention has shifted towards providing patients with optimal supportive care (Rosa *et al*. 2018, van der Molen *et al*. 2013, Zenga *et al*. 2018). As we move in the direction of health models driven by patient‐centred care, understanding the differential impacts of surgical procedures on subgroups of our patients is important to help improve our care models, patient education and support systems. Since the majority of patients undergoing this surgery are male, studies into total laryngectomy are typically male dominated. However, the proportion of female laryngectomees is rising; therefore, research should explore possible woman‐specific needs and issues in laryngectomees.

Quantitative studies investigating gender‐specific issues after total laryngectomy show that women might be at risk for a lower global health status, experience a greater impact on their relationship and have more stigma‐related problems (Cox *et al*. 2015, Lee *et al*. 2010, Offerman *et al*. 2015, Jansen *et al*. 2018). Graham and Palmer (2002) show that their studied group of female laryngectomees were younger at the time of surgery, were more likely to be working, more often had no partner, had a lower income, had more postoperative physical complaints, and relied more often on support from family and friends instead of support groups compared with their male counterparts. Women run a greater risk of a negative impact of total laryngectomy on their relationship than men after total laryngectomy (Offerman *et al*. 2015). Sexual problems and problems with intimacy after total laryngectomy are present in both male and female laryngectomees (Offerman *et al*. 2015, Singer *et al*. 2008). Offerman *et al*. (2015) demonstrate that women experience more sexual problems than their male counterparts do following total laryngectomy. Furthermore, these female laryngectomees report that there is less openness in discussing the consequences of their condition in the family (Offerman *et al*. 2015). It is shown that women have a lower global health status and quality of life after total laryngectomy (Lee *et al*. 2010). The procedure might impact women more in social functioning than men because of the lower pitched voice and altered appearance (Cox *et al*. 2015).

Qualitative research can contribute in gaining insight in women‐specific needs since it highlights issues and needs from the patient's perspective. However, the number of qualitative studies into the daily impact of total laryngectomy is still limited (Bickford *et al*. 2013, Dooks *et al*. 2012, Mertl *et al*. 2018, Noonan and Hegarty 2010). Existing studies focus on daily functional and psychological difficulties of patients, demonstrating that they struggle with the cancer diagnosis, psychological issues (e.g., anxiety, depression), and adjusting to life after total laryngectomy. Because this body of literature predominantly features the perspective of men, little is known about experiences specific to women. Gardner (1966) first describe the impact of total laryngectomy in affected women. Their focus on proper wifely duties and attitudes is fairly outdated, although it does confirm the presence of gender‐specific issues. Dooks *et al*. (2012) present a single female participant in their qualitative study as a contrast case, since she was the most tearful and depressed. Bickford *et al*. (2013) suggest that women after total laryngectomy have specific needs, since one of the female participants in their qualitative study indicates that she would appreciate having contact with other young female laryngectomees. Several authors state that future research should address the specific issues and needs of women (Bickford *et al*. 2013, Cox *et al*. 2015, Dooks *et al*. 2012).

Addressing this gap in the literature, the present paper aims to provide insight into the everyday impact of total laryngectomy on women, while examining their specific rehabilitation needs. This study uses a qualitative approach to investigate women's experiences following total laryngectomy, with a special focus on the period surrounding the procedure and long‐term functional and psychosocial outcomes.

## Methods

This paper is based on a qualitative descriptive study focusing on women's experiences following total laryngectomy. Interpretative phenomenological analysis as a qualitative approach is used in order to explore and understand the experience of a particular phenomenon, in this case experiences of women living with total laryngectomy. Braun *et al*.'s method for thematic analysis was used as a guideline to structure the analysis process (Braun *et al*. 2014).

Informed consent was obtained from all participants included in the study. The study was registered in the Netherlands under registration number METC17.1655/P17LTL.

After obtaining ethical approval, possible participants were selected from [The Netherlands Cancer Institute]. Inclusion criteria were: female; the total laryngectomy was at least 1 year ago; and they did not have recurrent disease. There were 32 possible candidates. A maximum sampling strategy was used to include a diverse group of women in terms of, for instance, relationship status, number of years since the total laryngectomy and ethnicity. Another consideration was to include participants who had acted as a patient visitor. Eight candidates were approached for the study. They were provided with the participant information form, and contacted 1 week later to determine intent to participate. All agreed to participate. The number of participants was determined by saturation of the data. Participants were between 60 and 77 years old and were operated 1–31 years ago. Two participants had undergone total laryngectomy as a primary treatment; three as a salvage procedure; and three due to a dysfunctional larynx after previous treatment. All used a voice prosthesis to communicate. Seven participants were able to speak in fluent sentences; one participant was limited in her verbal communication and had a poor intelligibility. One participant needed nutritional support; and one was limited in her verbal communication and also partly tube feeding dependent. At time of the interview, six respondents had a partner, one was single and one was a widow (see table 1 for participants’ characteristics).

**Table 1:** Participants included in the study

Participant	Indication total laryngectomy (TL)	Age at TL (years)	Age at time interview (years)	Partner	Type of speech	Intelligibility	Diet	Tube feeding	Highest education	Working before TL/at time of interview
1	Salvage	67	68	Yes	TES	Good	Oral intake, avoid specific food, nutritional support	No	Secondary education	No/no
2	Dysfunctional larynx	71	74	Yes	TES	Poor	Oral intake, soft diet	Yes	University	No/no
3	Salvage	54	67	Yes	TES	Good	Oral intake, normal diet	No	Higher vocational education	Yes/no
4	Primary	47	65	No	TES	Good	Oral intake, avoid specific food,	No	Higher vocational education	Yes/yes
5	Dysfunctional larynx	69	74	Yes	TES	Good	Oral intake, avoid specific food	No	Vocational education	No/no
6	Dysfunctional larynx	52	76	Yes	TES	Good	Oral intake, avoid specific food	No	Lower education	Yes/no
7	Salvage	29	60	Yes	TES	Good	Oral intake, normal diet	No	Secondary education	Yes/yes
8	Primary	47	62	No^a^	TES	Good	Oral intake, normal diet	No	Vocational education	Yes/yes

Notes: ^a^Participant was married during and after the period of TL, but has been a widow for 1 year.

TL, total laryngectomy; TES, tracheo‐oesophageal speech.

The interviews took place between December 2017 and March 2018. They were conducted at the respondents’ private homes and lasted around 90 min. Before the start of the interview, participants signed informed consent forms. In four cases the partner was also present during the interview.

The interviews were mostly conducted by two interviewers ((alternating KS, AK and GY). KS MSc works as a speech therapist and PhD student and has a background in health sciences. AK MSc works as a speech therapist and junior researcher and has a background in health sciences. KS and AK were already familiar with some of the participants due to their clinical work as speech therapists. MB MD PhD is a head and neck surgeon. LM PhD works as a postdoctoral fellow and speech therapist. GY PhD works as a postdoctoral fellow and has a background in medical humanities and qualitative methods in healthcare research.

The interviews were conducted using an interview guide, a so‐called semi‐structured approach (see appendix A). The guide was developed by deriving topics from the existing qualitative literature (Bickford *et al*. 2013, Dooks *et al*. 2012, Noonan and Hegarty 2010) and reviewed by an expert panel of healthcare professionals. A Roland Edirol digital recorder and Logitech HD Webcam C510 was used to obtain audio and video recordings of the interviews. Video recordings were used to support intelligibility in case of poor voice outcomes. All interviews were transcribed verbatim. After each interview, participants were asked whether they would be willing to answer follow‐up questions via email. All eight participants agreed; three were subsequently approached; two of them gave a written response.

**Figure 1:**
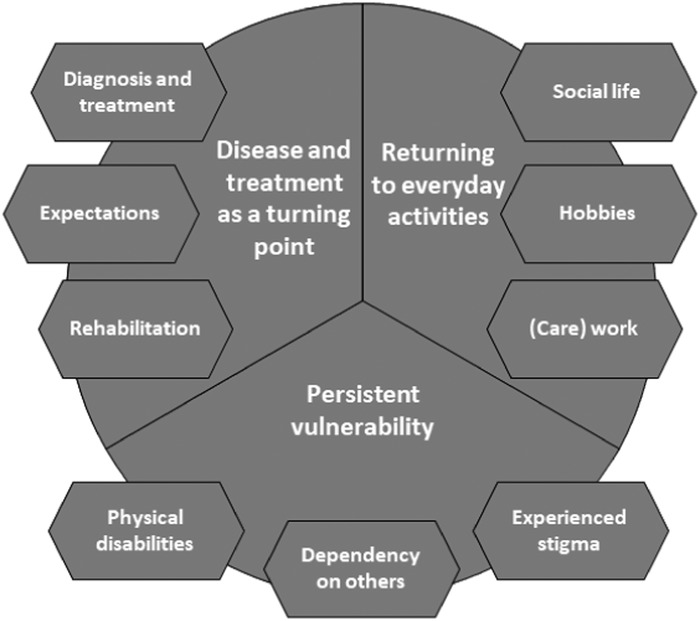
Three main themes and sub‐themes that resulted from the analysis of the interviews.

The interview data were analysed following the six phases described in the method of thematic analysis (Braun *et al*. 2014), while also taking interpretative phenomenological analysis as a lead (Smith *et al*. 2009). This method for the analysis of interviews emphasizes how respondents make sense of their own subjective experiences, in particular those following a life‐changing event. In accordance with both methodological approaches, the first step includes familiarization with the data by repeatedly reading the transcriptions. In addition, the interviewers all wrote short reflection reports following each interview (phase 1; Braun *et al*. 2014). Coding and analysis were initiated by highlighting potential codes together. Weekly meetings were held to discuss project's progress, initial coding and the analysis (phase 2; Braun *et al*. 2014). Next, members of the project team each coded a share of the interviews, while also regularly discussing the coding process together. After going through the first two interviews by hand, NVivo software (v11) was used to structure the coding process, creating a long list of codes along the data set. Inter‐coder reliability was monitored by keeping a master list of all codes, tracking all changes and regularly discussing these. Next, in an iterative process encompassing several joint sessions, codes were sorted into potential themes (phase 3; Braun *et al*. 2014). To visualize relationships between codes and themes, a ‘mind map’ was created (phase 4; Braun *et al*. 2014). This process resulted in a set of three fully worked‐out themes, each encompassing three sub‐themes (figure 1) (phase 5; Braun *et al*. 2014). Themes cover recurring issues, as well as issues that respondents indicated were meaningful to them. The themes and categories were checked against the entire data set by [acronym]. The report of the analysis served as a basis for the present paper (phase 6; Braun *et al*. 2014). After finishing the first draft of the report, a two‐page summary was created to perform participant checking. The participants received this summary by mail and were invited to respond via a response form or telephone. No comments were provided by the participants.

## Results

Three main themes were identified: ‘Disease and treatment as a turning point’, ‘Re‐establishing meaningful everyday activities’ and ‘Persistent vulnerability’ (figure 1). This section discusses each theme. The first theme is presented more briefly due to an overlap with existing qualitative literature on the experiences of individuals undergoing total laryngectomy (Bickford *et al*. 2013, Dooks *et al*. 2012, Noonan and Hegarty 2010).

### Disease and treatment as a turning point

This first theme highlights the fact that respondents experienced their disease and its treatment as a turning point in their lives. This shift already started before the procedure, when participants were first confronted with symptoms and functional issues, received their diagnosis, and were offered a treatment plan. When presented with the treatment plan, some respondents initially felt an aversion to total laryngectomy:

I used to say: ‘I'll have everything [medically necessary] done but not that.’ Because I had seen people [with total laryngectomy] walk around in the hospital, and then I said: ‘That's not for me.’ I'm not going to walk around with a hole in my throat. (Participant 5)

Some needed to go into surgery quickly, which was distressing and required last‐minute practical arrangements. One participant spoke about the impact the procedure had on her family life; her adolescent children and partner had to make a lot of effort to combine normal family life, school and work with visiting the hospital and caring for their mother/wife.

Participants’ expectations regarding the surgery and its outcome were shaped by the preoperative counselling they received in the hospital, information provided by patient visitors (a laryngectomized individual) and information they had sought themselves. Two respondents said the counselling they received from the hospital was too technical; they missed receiving information about the daily implications of having a total laryngectomy. All participants remembered their preoperative meeting with the laryngectomized patient visitor vividly. Many were relieved to meet someone who had undergone the surgery, and could still speak, eat and function well. This provided a positive, hopeful image of life after total laryngectomy. Three respondents stated that this meeting convinced them to undergo the procedure. However, some respondents indicated that, with hindsight, the laryngectomized patient visitor had provided an overly positive image of the surgery's outcome and that they neglected to discuss ongoing difficulties. Most emphasized the importance of meeting an individual who had already undergone the surgery to obtain an understanding of the procedure's outcomes (e.g., voice, functioning).

During their stay in the hospital and the first period at home, respondents experienced challenges in adjusting to their altered body. After the surgery, when recovering on the hospital's ward, they were not able to speak. To compensate, respondents used mouthing, gestures and writing to communicate. They indicated that being unable to speak can be severely distressing because it interferes with the capacity to communicate one's concerns clearly and in real time. Before and during standard medical procedures (e.g., suctioning mucus out of a tracheostoma), participants experienced little opportunity to ask questions, and their voiceless expressions of discomfort were brushed aside. Learning to speak again was often a physical and emotional challenge. All respondents disliked the sound of their new voice; they described their voices as ‘unfeminine’, ‘unpleasant,’ ‘gruff’, ‘scratchy’ or ‘grumpy’. Functional issues respondents mention included reduced physical fitness, the need for use of medical aids, swallowing difficulties, and managing mucus discharge from the tracheostoma.

### Returning to everyday activities

The second theme captures respondents’ experiences as they tried to return to everyday activities after recovering sufficiently. Their re‐integration into everyday life took place in three main domains: social life, hobbies and (care) work.

### Social life

Respondents described the impact of total laryngectomy on the relationship with their partner. Married respondents mostly experienced their husband as helpful and understanding, although they described becoming more dependent on their spouse, as the total laryngectomy reduced their ability to fulfil care activities such as cooking and looking after (grand)children. This led to shifts in the relationship's give‐and‐take balance, alterations in familiar role patterns, and changes in expectations between laryngectomee and partner:

I came home quite disabled, […] I couldn't speak well [and experienced] much coughing. [So, my husband] really thought: ‘my God, I'm stuck with an old woman’. […] At one point I thought: I am going to leave him. […] I refused to be a wife who is just being tolerated. […] Like some kind of glorified housemaid, you know. No way. Take it or leave it. (Participant 7)

Three respondents discussed their experiences regarding sexual intercourse. One respondent started having intercourse again after bringing this subject up with her husband; she stated that she needed intimacy. Two respondents no longer had sexual intercourse after total laryngectomy. One indicated that she did not feel comfortable having sex because of her changed voice and the risk of coughing. The single participant had her total laryngectomy at the age of 45 and did not find a partner after surgery. She started to date after her rehabilitation period. Before the procedure, she experienced being quite popular with the opposite sex. After being rejected repeatedly because of her voice after the surgery, she stopped dating.

One respondent gave birth to two children after her surgery. She discussed her wish to conceive with her specialist, since she worried about her physical abilities (e.g., push during labour). In hindsight, she indicates that she did not experience any problems from her condition during this period, except for the voice prosthesis which was dislocated due to hormonal changes.

Participants experienced changes in social contact with unfamiliar people. Participants frequently experienced negative reactions or assumptions regarding their voice, in direct interactions as well as over the telephone. Many respondents discussed being self‐conscious about their appearance and how they present themselves to others, by covering their stoma, for example. Some mentioned listeners associate the sound of their altered voice with being grumpy or interpret it as masculine. As one participant stated:

[When I would] telephone the post office about a delivery, [they'd say]: ‘Sir, stop complaining.’ And then when I would come pick it up, they would say: ‘Oh ….’ They always think I am a man [before seeing me]. […] People [also] treat me gruffly. Then, when they see me in real life, they suddenly become very friendly. (Participant 4)

Among family and friends, participants often experienced understanding and support. Nevertheless, these intimates also needed to get used to the alterations following total laryngectomy. In particular, respondents’ new method of speaking, which is slower, less clear, and less loud, affected their ability to interact with others. All respondents experienced limitations in social situations that require shouting, whispering, singing, reading out loud, providing quick retorts, or expressing emotions vocally (e.g., laughing, crying). Consequently, some participants eventually withdrew from social situations that involved groups:

[Communicating] is different when everyone is over here [at my house] for my birthday. You barely hear me talking then. I'll just be sitting there, watching everything. It is harder for me to be intelligible [in a group]. [So] I just don't [speak]. (Participant 1)

The total laryngectomy also led to new social contacts, especially with peers (individuals who had undergone the same surgery). The respondent who had her surgery at age of 29 realized that she could not relate to the experience of elderly laryngectomized individuals when she visited a patient meeting:

I went [to a patient meeting] twice. First of all […], they were all much older than me. Discussions were like: ‘My grandchildren will not hear my own voice.’ And I thought: ‘Well, come on, my children have never heard my voice. You know what, [this setup] didn't fit my experiences.’ (Participant 7)

### Hobbies

To take up their former hobbies, participants either made practical adjustments so they could still perform these after total laryngectomy or took up new leisure activities. One respondent modified her boat to decrease the risk of falling into the water. Many participants developed new interests and hobbies that would be easy to engage in despite their condition (e.g., sewing clothes, making necklaces to cover the stoma, gardening). Two participants joined a special choir for head and neck cancer patients. This enabled them to enjoy singing and performing and to have fun with their voice again.

### (Care) work

Four respondents had a job before the total laryngectomy, but none of them was able to return to it following their surgery since these positions required much speaking (e.g., teacher, secretary, saleswoman). Two participants actively sought a job after their total laryngectomy and got rejected repeatedly during the application process.

The first years after my laryngectomy I still applied [for vacancies] […]. The first years, I thought [my condition] wasn't too bad. I didn't realize how severe it was. How many disabilities I had […]. After five or six years I stopped [applying], thinking: ‘This isn't going to happen.’ (Participant. 4)

All respondents of working age were declared unfit to work. Nevertheless, five respondents started work‐related activities. Some started volunteering in their community or within the Dutch organization for head and neck cancer patients (e.g., patient–visitor, providing lessons). They valued their ability to help others by sharing their own experiences. Others set up their own business in order to remain financially secure; one provided painting lessons to small groups, and one started her own bed and breakfast.

Most participants were still able to perform (some) informal care‐work, including household activities and taking care of others. Nevertheless, those who required assistance from their husband, relatives or a household help reported they had difficulties transitioning from being the care provider to being its recipient. They found it hard to delegate or outsource tasks such as cooking or cleaning:

Taking care of the household and not wanting to delegate [tasks] […] is really very difficult for me. […] I now need a whole week for things that previously took me one day to accomplish. (Participant 5)

Respondents who regularly took care of children, especially (grand)mothers, mentioned that it was difficult for them to warn a child in a dangerous situation, and experienced challenges while singing or reading books. Just calling out to capture a child's attention in public also proved potentially embarrassing—and therefore more challenging:

And with my kids, […] at the shops, I would clap my hands and then they'd know: ‘Oh, mama is calling.’ Because I didn't want to [shout] then, in such a shopping mall. […] You really don't want to stand out [with your unusual voice], then. (Participant 7)

### Persistent vulnerability

The third theme identified in the interviews was persistent vulnerability. Although participants found various ways to return to the daily activities, they experienced vulnerability, including physical disabilities, dependence on others for support and stigma.

### Physical disabilities

Participants were confronted with several ongoing physical changes in breathing, swallowing, speaking and appearance. The physical limitations of the tracheo‐oesophageal voice made respondents apprehensive about situations where they were dependent on their verbal abilities. One participant noted that she feared being unable to call for help during an emergency due to living alone:

It is a lot harder when you're single; […] being unable to talk is very frightening […] when you're home alone and thinking that you're choking or something. (Participant 4)

All respondents reported consciousness of dealing with water in daily settings, such as being on a boat, cycling near open water or taking a shower.

### Dependency on others

Owing to the changes after total laryngectomy described above, some participants reported a continuous dependency on their social network and on healthcare. In some situations, such as using the telephone, participants needed family members or partners to speak for them. One participant spoke about the support she required in caring for her young children:

When we would go swimming with [my] kids, I always took a friend with me, because well, I had to be able to send someone into the water [if necessary] since I can't do it myself. (Participant 7)

Some participants received financial support from relatives. Others remained dependent on family members and friends for transport to the hospital or other forms of medical care.

Respondents reported a dependence on healthcare as well, since they kept requiring medical support, for example, managing problems with the stoma and the voice prosthesis. When travelling, participants preferred being treated in a specialized centre, since healthcare professionals are not always familiar with total laryngectomy. For several respondents, this all meant they were hesitant to leave town or go abroad.

### Experienced stigma

Participants reported experiencing stigma regularly, especially in the form of unwanted attention: other people staring at their stoma, making (offensive) comments or asking (intrusive) questions. One participant shared her experiences of being bullied by neighbourhood children:

[…] Children ringing the doorbell, [shouting]: ‘Witch, witch, you cannot talk!’ […] ‘Say something, say something!’ […] To me that is awful. (Participant 4)

The respondents assumed that this unwanted attention resulted from other people's unfamiliarity with total laryngectomy. Managing unwanted attention was bothersome or difficult for some participants.

When asked directly, most participants indicated that a total laryngectomy impacts men and women in similar ways due to the physical issues involved. Nevertheless, some respondents proposed that the challenges affected women face in daily life are bigger, especially in social situations. They presumed women speak more and are judged on their appearance and voice more than men. Participants gave examples of women‐specific stigma they had experienced, including being taken for a man over the telephone and receiving comments about how they look or about their low‐pitched voice.

## Discussion

As the stories of women with total laryngectomy reveal, both their disease and its treatment formed *a turning point* in their lives. Respondents made efforts to re‐integrate into the social communities they belonged to before their surgery and *return to former everyday activities*. All found various ways to participate in daily life again. Nevertheless, participants remain *persistently vulnerable* after total laryngectomy, because of the effort required by foreseeing and managing functional, social and health‐related issues. As a result, participants are required to continuously manage their disabilities as well as other people's responses to them. The disease and its treatment mean that both body and self are radically and irrevocably altered.

This study explored the perceptions of females on their life after total laryngectomy and shows the presence of women‐specific issues. Interestingly, when asked directly, most participants did not think there were substantial differences between the experiences of men and women after total laryngectomy. Similarly, Graham and Palmer (2002) found that responses of men and women after total laryngectomy were more similar than dissimilar. Lee *et al*. (2010) showed that females following total laryngectomy had significantly lower global health status than males and lower levels of physical, emotional, cognitive and social functioning. Our findings illustrate that some of the challenges participants experience are due to their inability to adhere with societal expectations regarding feminine roles and activities (e.g., taking care of others and the home, looking attractive, having a high pitched voice). Many respondents discussed being self‐conscious about their appearance and how they present themselves to others. In addition, they all disliked the sound of their voice, which several described as unfeminine. Our findings herein therefore corroborate Cox *et al*.’s (2015) hypothesis that changes in voice, sound, and speech quality after total laryngectomy lead to a loss of femininity.

The present study also demonstrates that the relationship of women with their partner is influenced by the total laryngectomy. Cancer treatment in general impacts the spousal relationship (Li and Loke 2014, Pitceathly and Maguire 2003). Offerman *et al*. (2015) showed that female laryngectomees run a greater risk of a negative impact of total laryngectomy on their relationship than their male counterparts. Specifically, the present results show that the surgery and its aftermath leads to a noticeable shift in give‐and‐take balance between partners. This shift is presumably associated with the fact that women in a heterosexual relationship still mainly perform the majority of household chores (Powell and Greenhaus 2010, Shelton and John 1996).

In line with this point, this study also revealed women‐specific issues in participants’ performance of informal care‐work. Some respondents found it hard to depend on others who cared for them or delegate household chores after the surgery. Problems with informal care‐work were also a recurring topic in interviews for participants who were (grand)mothers. Earlier studies on the impact of total laryngectomy on affected individuals did not investigate how care‐work activities take shape in daily life after the procedure. Again, since the majority of childcare is still performed by women (Folbre 2006), (informal) care‐work is a domain in which gender differences are still very much relevant. To further investigate the impact of total laryngectomy on women therefore required addressing both shifts in the spousal relationship and care‐work.

Issues pertaining to femininity also arose in the areas of intimacy and sexuality. Research shows that libido and sexual problems are common after treatment for laryngeal cancer in both male and female patients (Singer *et al*. 2008). Singer *et al*. (2008) showed that having reduced physical strength (‘not enough stamina’) rather than the changes in appearance and/or the sputum are considered as most problematic for having sexual intercourse. By contrast, the present study suggests that the issues women experience herein involve both their altered appearance and functioning, that is, physical disabilities. Thus, our respondents worried that the noises their stoma produces and their coughing fits might be unattractive to their partner or interfere with the intercourse. Similarly, Offerman *et al*.’s (2015) study on the impact of total laryngectomy on the spousal relationship showed that female laryngectomees experience more deterioration in their sexual relationship and more sexual problems when compared with their male counterparts. Further research should explore the role of appearance, functionality and physical strength in reduced sexuality in male as well as female patients.

The women in the present study who were of working age could not return to their former job after surgery. After a period of recuperation and adaption, most wanted to re‐integrate into work activities nevertheless. In the end, two respondents started a business, and three turned to volunteer work (one combined the two). In Graham and Palmer (2002), the female laryngectomy participants included were younger at the time of surgery. As a consequence, more females compared with males were working or on disability leave after surgery (Graham and Palmer 2002). This highlights the importance of returning to work‐related activities for women following total laryngectomy. Wells *et al*.’s (2013) systematic review of qualitative studies exploring return to work after cancer demonstrates that successfully returning to work depends on shifts and adjustments in each aspect of what is already a complex set of factors at the individual, organizational and societal level (Wells *et al*. 2013). Successful return to work after total laryngectomy might be influenced by former work activities, functional outcomes and adjustment, and age at total laryngectomy. As the present results suggest, it also depends on gender. Our respondents—and women generally—are still predominantly employed in areas such as teaching, administration or care, which involve communication and service orientation.

All eight respondents in this study have (had) contact with peers. Peer support is highly satisfying for people with cancer (Hoey *et al*. 2008, Watson 2017). Peer support provided by a laryngectomized patient visitor can play an important role in both information giving and counselling before surgery (Fitzgerald and Perry 2016, Raol *et al*. 2017, van der Molen *et al*. 2013). Indeed, all eight participants of this study remembered the preoperative meeting with a laryngectomized patient visitor vividly. Although the female participants of the present study did not explicitly indicate that they would have liked to meet a female patient, we suggest such a match might aid prospective women laryngectomees form realistic expectations concerning voice quality, appearance, and other outcomes that may affect women differently than men. Similarly, one respondent who received her surgery at age 29 indicated she could not relate to elderly peers. Earlier research, too, shows that younger female laryngectomees prefer contact with younger peers (Bickford *et al*. 2013).

### Recommendations for clinical practice

The insights provided by this study may help improve clinical practice by enabling a more patient‐centred approach in pretreatment counselling and rehabilitation. As we move towards health models driven by patient‐centred care, understanding the differential impacts of surgical procedures on subgroups of our patients, in this case women, is critically important to help improve our care models, patient education and support systems. We support the conclusions of earlier research recommending personalized rehabilitation provided by a multidisciplinary team and recognize the presence of unmet supportive care needs in women undergoing total laryngectomy (Bickford *et al*. 2013, 2018, Eadie *et al*. 2018, Perry *et al*. 2015, Passchier *et al*. 2016, Lee *et al*. 2010, Sharpe *et al*. 2019, Jansen *et al*. 2018).

First, we recommend that standard pretreatment counselling procedures be re‐evaluated. Counselling involves exploring patients’ priorities, values and concerns about the treatment and the outcome, as well as possible benefits, risks and implications of the treatment options (Laccourreye *et al*. 2012, Raol *et al*. 2017, Fitzgerald and Perry 2016). However, we recommend that the information given also includes expected everyday issues after the procedure, specifically as experienced from a patients’ point of view. This implies providing information on gender‐specific issues. It therefore seems prudent to match patients and patient visitors based on both gender and age to facilitate realistic expectations and enhance peer support possibilities.

Second, we recommend tailored rehabilitation programmes following total laryngectomy, with attention to gender‐specific issues. The present study suggests that women face specific problems while reintegrating on an individual as well as on a societal level after total laryngectomy. Lee *et al*. (2010) propose that women may particularly benefit from rehabilitation programmes which aim to improve emotional and social functioning post‐laryngectomy, since they found this to be significantly impacted in female laryngectomees. The present study shows the persistent vulnerability on the long term including the dependence on others, stigma‐related problems, and problems with returning to the former job. Thus, rehabilitation programmes should include psychosocial and practical support to pursue participation in social activities and work‐related activities.

Third, it is important to involve the partner in the treatment and rehabilitation process. Women following total laryngectomy experience a greater impact of the procedure on their relationship than men (Offerman *et al*. 2015). The present study specifies this with the reported shift in give‐and‐take balance of the spousal relationship, challenges in performing care‐work and problems with intimacy. We therefore highlight the importance of involving partners in the process of rehabilitation and recommend that clinicians create openness in discussing the impact of the surgery on the relationship and family roles. This will allow females following to re‐integrate into their former roles in as well family, social life as in work.

This study has some limitations. Qualitative research in general is never representative for the entire population, because it draws on the experiences of a relatively small sample. Also, since only women were recruited, experiences specific to men were not addressed in this study. However, the main themes ‘Disease and treatment as a turning point’, ‘Returning to everyday activities’ and ‘Persistent vulnerability’ are likely to occur with men as well. Furthermore, the study necessarily included participants who had a relatively high level of speaking proficiency with their voice prosthesis and were therefore able to express themselves verbally. Despite this bias, our findings illustrate a wide range of issues beyond communication. This paper therefore provides an impression of an extensive range of possible issues women may be confronted with after total laryngectomy.

## Conclusions

The accounts provided by the women interviewed in this study show that undergoing total laryngectomy has a major impact on their everyday life. The disease and its treatment form a turning point in their lives, after which they experience challenges in while returning to everyday activities and persistent vulnerability. Women‐specific issues are present in returning to work, the performance of informal care‐work, the spousal relationship, intimacy and social interaction (mostly stigmatization due to voice and appearance). The aim of this study was to reveal women‐specific issues, but we nevertheless assume that some of the reported issues are present for men as well. Based on the findings of this study it is recommended to include information on changes in daily functioning in the pretreatment counselling procedures, and the contribution of an aged‐ and gender‐matched patient visitor would be valuable. Tailored rehabilitation for women following total laryngectomy is recommended to enhance participation in social roles and work‐related activities. Within this process of treatment and rehabilitation, it is important to involve the partner in order to discuss the impact of the surgery on the relationship and family roles. To understand fully the gender‐specific aspects of total laryngectomy, future research should focus on the particular ways in which the procedure affects both women and men in specific areas of daily life (e.g., returning to work, care activities and intimate relationships).

## Appendix A Interview guide

Medical history

- Diagnosis, information, treatment & rehabilitation process
- Memories, what made a big impression

First time voicing

- Expectations
- Reactions: contact with family, friends and unfamiliar people)
- Take me along on a normal day, what role does the total laryngectomy play in your day?

Voice use & sound

- Interactions (acquaintances/strangers, children/adults, changed relationships)
- Situations, personal experience, functionality of the voice
- Expression of emotions
- Femininity of the voice
- Does the voice suit you?

Changed appearance (interactions, situations, personal experience, visibility)

Use of medical aids, use of body, everyday functioning

- Care, stoma, medical aids, hand use for voice

Functional & physical issues

- Daily activities, sports, nose, mouth, throat, lungs, stoma, physical fitness, fatigue, environmental conditions (temperature, noise)

Women‐specific issues

How do you look back on the choice of undergoing total laryngectomy?

Additional issues: Things we missed

## References

[jlcd12511-bib-0001] Bickford, J. , Coveney, J. , Baker, J. and Hersh, D. , 2013, Living with the altered self: a qualitative study of life after total laryngectomy. International Journal of Speech–Language Pathology, 15, 324–333. 23586580 10.3109/17549507.2013.785591

[jlcd12511-bib-0002] Bickford, J. M. , Coveney, J. , Baker, J. and Hersh, D. , 2018, Support following total laryngectomy: Exploring the concept from different perspectives. European Journal of Cancer Care (England), 27, e12848. 10.1111/ecc.12848 29671922

[jlcd12511-bib-0003] Braun, V. , Clarke, V. and Terry, G. , 2014, Thematic analysis. Qualitative Research in Clinical Health Psychology, 24, 95–114.

[jlcd12511-bib-0004] Cox, S. R. , Theurer, J. A. , Spaulding, S. J. and Doyle, P. C. , 2015, The multidimensional impact of total laryngectomy on women. Journal of Communication Disorders, 56, 59–75. 26186255 10.1016/j.jcomdis.2015.06.008

[jlcd12511-bib-0005] Dooks, P. , Mcquestion, M. , Goldstein, D. and Molassiotis, A. , 2012, Experiences of patients with laryngectomies as they reintegrate into their community. Supportive Care in Cancer, 20, 489–498. 21298450 10.1007/s00520-011-1101-4

[jlcd12511-bib-0006] Eadie, T. , Kapsner‐Smith, M. , Bolt, S. , Sauder, C. , Yorkston, K. and Baylor, C. , 2018, Relationship between perceived social support and patient‐reported communication outcomes across communication disorders: a systematic review. International Journal of Language and Communication Disorders, 53, 1059–1077. 30039920 10.1111/1460-6984.12417 PMC7335018

[jlcd12511-bib-0007] Fitzgerald, E. and Perry, A. , 2016, Pre‐operative counselling for laryngectomy patients: a systematic review. Journal of Laryngology & Otology, 130, 15–20. 26567459 10.1017/S0022215115002984

[jlcd12511-bib-0008] Folbre, N. , 2006, Measuring care: Gender, empowerment, and the care economy. Journal of Human Development, 7, 183–199.

[jlcd12511-bib-0008a] Gardner, W. H. , 1966, Adjustment problems of laryngectomized women. Archives of Otolaryngology, 83, 31–42. 5900434 10.1001/archotol.1966.00760020033011

[jlcd12511-bib-0009] Graham, M. S. and Palmer, A. D. , 2002, Gender difference considerations for individuals with laryngectomies. Contemporary Issues in Communication Science and Disorders, 29, 59–67.

[jlcd12511-bib-0010] Hoey, L. M. , Ieropoli, S. C. , White, V. M. and Jefford, M. , 2008, Systematic review of peer‐support programs for people with cancer. Patient Education and Counseling, 70, 315–337. 18191527 10.1016/j.pec.2007.11.016

[jlcd12511-bib-0011] Jansen, F. , Eerenstein, S. E. J. , Lissenberg‐Witte, B. I. , Uden‐Kraan, C. F. V. , Leemans, C. R. and Leeuw, I. M. V. D. , 2018, Unmet supportive care needs in patients treated with total laryngectomy and its associated factors. Head and Neck, 40, 2633–2641. 30462875 10.1002/hed.25358

[jlcd12511-bib-0012] Laccourreye, O. , Malinvaud, D. , Holsinger, F. C. , Consoli, S. , Ménard, M. and Bonfils, P. , 2012, Trade‐off between survival and laryngeal preservation in advanced laryngeal cancer: the otorhinolaryngology patient's perspective. Annals of Otology, Rhinology and Laryngology, 121, 570–575. 10.1177/000348941212100902 23012894

[jlcd12511-bib-0013] Lee, M. T. , Gibson, S. and Hilari, K. , 2010, Gender differences in health‐related quality of life following total laryngectomy. International Journal of Language and Communication Disorders, 45, 287–294. 20131961 10.3109/13682820902994218

[jlcd12511-bib-0014] Li, Q. and Loke, A. Y. , 2014, A literature review on the mutual impact of the spousal caregiver–cancer patients dyads: ‘communication’, ‘reciprocal influence’, and ‘caregiver–patient congruence’. European Journal of Oncology Nursing, 18, 58–65. 24100089 10.1016/j.ejon.2013.09.003

[jlcd12511-bib-0015] Mertl, J. , Zackova, E. and Repova, B. , 2018, Quality of life of patients after total laryngectomy: the struggle against stigmatization and social exclusion using speech synthesis. Disability and Rehabilitation: Assistive Technology, 13, 342–352. 28447495 10.1080/17483107.2017.1319428

[jlcd12511-bib-0016] Noonan, B. J. and Hegarty, J. , 2010, The impact of total laryngectomy: the patient's perspective. Oncology Nursing Forum, 37, 293–301. 20439213 10.1188/10.ONF.293-301

[jlcd12511-bib-0017] Offerman, M. P. , Pruyn, J. F. , De Boer, M. F. , Busschbach, J. J. and Baatenburg De Jong, R. J. , 2015, Psychosocial consequences for partners of patients after total laryngectomy and for the relationship between patients and partners. Oral Oncology, 51, 389–398. 25631352 10.1016/j.oraloncology.2014.12.008

[jlcd12511-bib-0018] Passchier, E. , Stuiver, M. M. , Van Der Molen, L. , Kerkhof, S. I. , Van Den Brekel, M. W. and Hilgers, F. J. , 2016, Feasibility and impact of a dedicated multidisciplinary rehabilitation program on health‐related quality of life in advanced head and neck cancer patients. European Archives of Oto‐Rhino‐Laryngology, 273, 1577–1587. 26024692 10.1007/s00405-015-3648-z

[jlcd12511-bib-0019] Perry, A. , Casey, E. and Cotton, S. , 2015, Quality of life after total laryngectomy: functioning, psychological well‐being and self‐efficacy. International Journal of Language and Communication Disorders, 50, 467–475. 25703153 10.1111/1460-6984.12148

[jlcd12511-bib-0020] Pitceathly, C. and Maguire, P. , 2003, The psychological impact of cancer on patients’ partners and other key relatives: a review. European Journal of Cancer, 39, 1517–1524. 12855257 10.1016/s0959-8049(03)00309-5

[jlcd12511-bib-0021] Powell, G. N. and Greenhaus, J. H. , 2010, Sex, gender, and decisions at the family → work interface. Journal of Management, 36, 1011–1039.

[jlcd12511-bib-0022] Raol, N. , Lilley, E. , Cooper, Z. , Dowdall, J. and Morris, M. A. , 2017, Preoperative counseling in salvage total laryngectomy: content analysis of electronic medical records. Otolaryngology–Head and Neck Surgery, 157, 641–647. 28828922 10.1177/0194599817726769

[jlcd12511-bib-0023] Rosa, V. M. , Fores, J. M. L. , Da Silva, E. P. F. , Guterres, E. O. , Marcelino, A. , Nogueira, P. C. , Baia, W. R. M. and Kulcsar, M. A. V. , 2018, Interdisciplinary interventions in the perioperative rehabilitation of total laryngectomy: an integrative review. Clinics, 73(Suppl. 1), e484s. Epub September 06, 2018. 10.6061/clinics/2018/e484s PMC6113851 30208167

[jlcd12511-bib-0024] Sharpe, G. , Costa, V. C. , Doubé, W. , Sita, J. , Mccarthy, C. and Carding, P. , 2019, Communication changes with laryngectomy and impact on quality of life: a review. Quality of Life Research, 28, 863–877. 30417205 10.1007/s11136-018-2033-y

[jlcd12511-bib-0025] Shelton, B. A. and John, D. , 1996, The division of household labor. Annual Review of Sociology, 22, 299–322.

[jlcd12511-bib-0026] Singer, S. , Danker, H. , Dietz, A. , Kienast, U. , Pabst, F. , Meister, E. F. , Oeken, J. , Thiele, A. and Schwarz, R. , 2008, Sexual problems after total or partial laryngectomy. Laryngoscope, 118, 2218–2224. 19029864 10.1097/MLG.0b013e318182cdc6

[jlcd12511-bib-0027] Smith, J. , Flowers, P. and Larkin, M. , 2009, Interpretative Phoneomological Analysis: Theory, Method and Research (London: SAGE).

[jlcd12511-bib-0028] Van Der Molen, L. , Kornman, A. F ., Latenstein, M. N. , Van Den Brekel, M. W. and Hilgers, F. J. , 2013, Practice of laryngectomy rehabilitation interventions: a perspective from Europe/the Netherlands. Current Opinion in Otolaryngology and Head and Neck Surgery, 21, 230–238. 23572017 10.1097/MOO.0b013e3283610060

[jlcd12511-bib-0029] Watson, E. , 2017, The mechanisms underpinning peer support: a literature review. Journal of Mental Health, 1–12. 10.1080/09638237.2017.1417559 29260930

[jlcd12511-bib-0030] Wells, M. , Williams, B. , Firnigl, D. , Lang, H. , Coyle, J. , Kroll, T. and Macgillivray, S. , 2013, Supporting ‘work‐related goals’ rather than ‘return to work’ after cancer? A systematic review and meta‐synthesis of 25 qualitative studies. Psycho–Oncology, 22, 1208–1219. 22888070 10.1002/pon.3148

[jlcd12511-bib-0031] Zenga, J. , Goldsmith, T. , Bunting, G. and Deschler, D. G. , 2018, State of the art: Rehabilitation of speech and swallowing after total laryngectomy. Oral Oncology, 86, 38–47. 30409318 10.1016/j.oraloncology.2018.08.023

